# Increasing gaps in health inequalities related to non-communicable diseases in South Australia; implications towards behavioural risk factor surveillance systems to provide evidence for action

**DOI:** 10.1186/s12889-018-6323-7

**Published:** 2019-01-08

**Authors:** Stefano Campostrini, Eleonora Dal Grande, Anne W. Taylor

**Affiliations:** 1 Department of Economics, Ca’ Foscari University of Venice, San Giobbe 873, 30121 Venice, Italy; 2 School of Medicine, The University of Adelaide, Adelaide, South Australia; 3 Population Research & Outcome Studies, Discipline of Medicine, The University of Adelaide, Adelaide, South Australia

**Keywords:** Behavioral risk factor surveillance, Chronic diseases, Non communicable diseases, Health inequalities, Trend analysis

## Abstract

**Background:**

Although Australia is a country cited as having generally low health inequalities among different socioeconomic groups, inequalities have persisted. The aim of this analysis was to highlight how inequalities have evolved over a 13 years period in South Australia (SA).

**Methods:**

Since 2002, over 600 interviews per month have been undertaken with SA residents through a computer assisted telephone survey method (total 77,000+). Major risk factors and chronic diseases have been analyzed providing trends by two socio-economic variables: education and a proxy of income (ability to save).

**Results:**

While income and educational gaps are reducing over time in SA, those that remain in the lower socio-economic groups have a generally higher prevalence of risk factors and chronic diseases. The health disparity gap is still relevant, although at a different extent, for all the variables considered in our study, with most appearing to be stable if not increasing over time.

**Conclusions:**

Surveillance can be a good source of information both to show the evolution of problems and to evaluate possible future interventions. Extensive effort is still required to “close the gap” of health inequalities in SA. More precisely targeted and properly implemented interventions are needed.

**Electronic supplementary material:**

The online version of this article (10.1186/s12889-018-6323-7) contains supplementary material, which is available to authorized users.

## Background

Australia is a country cited as having low health inequalities [1]. In 1994, health authorities in Australia published a report setting targets for better health outcomes [2]. The report highlighted the importance of monitoring different health outcomes between different socio-economic and demographic groups [2]. Although considerable attention has been paid to inequalities since the first national reports, and many calls for action have been made, health inequalities have persisted among different socioeconomic groups [3–5]. More recently, several interventions have been promoted, both at a public health and other government agency level. Notably in 2010, a document ‘Health in all Policies’, cited explicitly as a tool to tackle health disparities has been produced conjointly by the World Health Organization (WHO) and the Government of SA [6].

Although some positives changes have occurred in SA, Australia and worldwide (eg smoking), other targets are yet to be reached [5–7]. Relevant chronic disease and risk factor targets that require constant monitoring and assessment include harmful use of alcohol, physical inactivity, salt intake, tobacco use, diabetes and obesity [8].

The use of a behavioural risk factor surveillance (BRFS) system as a valuable source of information for inequalities monitoring has been advocated, particularly in relation to tracking trends [9, 10]. While some of the targets are not possible to monitor with the use of a BRFS system, others are. As such, we analyzed data from the South Australian Monitoring and Surveillance System (SAMSS) to test the most recent relevant trends on specific health disparities. The focus of these first analyses were to focus solely on trends, without at this stage, investigating possible causes, leaving this to future studies.

## Methods

### Data

SAMSS is owned by the South Australian Department of Health and Ageing (SA Health) and is an epidemiological monitoring system. SAMSS aims to detect and facilitate understanding of trends in the prevalence of chronic conditions, risk and protective factors, and other determinants of health. These data monitor departmental, state and national priority areas and are linked to key indicators such as state and national healthy weight targets [11].

Each month from July 2002, a sample of South Australians was randomly selected from the Electronic White Pages (EWP). Introductory letters were sent to each household selected to inform them of the upcoming telephone survey, and inviting the person who had the last birthday in the household to participate in a telephone interview. The interviews were conducted by professional interviewers, using Computer Assisted Telephone Interview (CATI) technology. Approximately 600 respondents participate in each SAMSS survey. Although data were collected on children, data presented in these analyses are for those aged 18 years and older. All data are weighted each month by sex, age, area of residence and probability of selection of the household using the latest Australian Bureau of Statistics (ABS) census data or estimated residential population data. Data were then raked to further adjust for weighting [12]. Data from July 2002 until June 2015 were utilised. Ethics approval for the survey was obtained from the ethics committee of the SA Health (HREC/14/SAH/200 & HREC/436.02.2014). The topics and questions included in SAMSS were developed in consultation with key personnel within SA Health, including relevant experts, and questions are based on previous work undertaken in Australian states and territories. Where possible, questions that had previously been included in other surveys, and which were perceived to ascertain reliable and valid data, were used or modified. Additional details on SAMSS methodology is available [11].

### Variables

The risk factors assessed were: overweight/obesity (calculated from self-reported height and weight and recoded into Body Mass Index (BMI) according to the WHO classification with BMI ≥ 25 nominated as unhealthy and BMI ≥ 30 classified as obesity) and fruit consumption (two or more serves per day, as suggested by National Health & Medical Research Council [13] and international guidelines [14].

We also examined the self-reported prevalence of chronic conditions, defined by answers to the question “have you ever been told by a doctor that you have… diabetes, asthma, heart disease, osteoporosis and/or arthritis”. Having any chronic condition (one or more) and multimorbidity (two or more) of these conditions were used in the analysis. Specific analyses were also undertaken for diabetes which is acknowledged as one of the chronic condition more sensitive to social determinants of health [15, 16].

Finally, we analysed self-reported mental health conditions, a combined variable created using positive answers to the question “Have you been told by a doctor that you have any of the following conditions (yes/no) in the last 12 months? The conditions were: anxiety, depression, stress related problems or any other mental health problem” and/or whether the respondent was currently receiving treatment for these conditions. Psychological distress was also measured by the Kessler 10 (K10) [17–19]. The K10 is a self-report, 10-item set of questions based on the level of anxiety and depressive symptoms experienced in the previous 4 weeks. The questions were scored to a single scaled item with respondents with high scores of 22–50 being classified as having high or very high levels of psychological distress.

To show possible disparities, we used the following two demographic variables: education (no school to secondary, trade certificate or diploma, degree or higher) and a question assessing “which best describes your money situation” (we spend more money than we get; we have just enough to get through to the next pay day; there’s money left over but we just spend it; we can save a bit now and then; we can save a lot) with the last two categories recoded as ‘able to save’. This question was used rather than household income as income has increased over this time period (in line with Consumer Price Index (CPI) increases).

### Statistical analysis

Analyses were conducted using chi-squared test for trend to detect differences in overall prevalence and for each level of educational attainment and household money situation for each risk factor and chronic condition between 2002/4 and 2013/15. Chi-squared tests were also undertaken to detect differences in prevalence of each risk factor and chronic condition by educational attainment and ‘ability to save’ for 2002/4 and 2013/15. All “don’t know” responses were treated as missing values. Annual prevalence over time for each of the risk factors and chronic conditions by educational attainment and ‘ability to save’ are presented graphically. The prevalence over time data were not standardized to a reference population. In total *n* = 74,127 interviews were conducted with adults aged 18 years or older. Response rates (RR) using the American association for Public Opinion Research [20] standards definition (AAPOR RR1) varied from 54.1 to 71.3% (mean = 65.5%).

Data were analysed using SPSS Version 20.0 (IBM SPSS Statistics, Armonk, NY, USA*)* and Stata (StataCorp, College Station, TX, USA) Version 13.0. All data presented were weighted to be reflective of the South Australian population using raking methodology by area (metropolitan/rural), age, gender, marital status, country of birth, educational attainment, and dwelling status (rented property vs other) to the most relevant South Australian population data (year), and probability of selection in the household.

## Results

The total number of interviews and response rate per year are included as Additional file 1: Table S1. In SA over the past 13 years the population have achieved higher levels of education (related to the fact that younger generations have generally higher level of education), with the proportion achieving a university degree or higher increasing from 12.6% (95% CI 12.0–13.2) to 18.7% (95% CI 18.2–19.3). In addition, the South Australian population are relatively richer with the ability to save increasing from 63.0% (95% CI 62.2–63.9) to 66.6% (95% CI 65.9–67.3) (Table 1).

**Table 1:** Socio-demographic situation, risk factors, chronic diseases and mental health comparison

	2002/4			2013/15			Cmp 2002/4 and 2013/15
	n	% (95% CI)	*P* value^a^	n	% (95% CI)	*P* value^b^	*P* value^c^
Demograpic Variables							
Educational attainment							
No schooling to secondary	7932	62.8 (62.0–63.6)		7969	47.7 (47.0–48.5)		< 0.001
Trade, certificate, diploma	3112	24.6 (23.9–25.4)		5602	33.5 (32.8–34.3)		
Degree or higher	1587	12.6 (12.0–13.2)		3132	18.7 (18.2–19.3)		
Household money situation							
Unable to save	4238	33.5 (32.7–34.3)		4654	27.8 (27.1–28.5)		< 0.001
Can save	7974	63.0 (62.2–63.9)		11,152	66.6 (65.9–67.3)		
Not stated	438	3.5 (3.2–3.8) *		930	5.6 (5.2–5.9)		
Risk Factors							
Overweight/Obesity	6487	54.9 (54.0–55.8)		9570	60.8 (60.1–61.6)		< 0.001
No schooling to secondary	4104	56.1 (55.0–57.2)	< 0.001	4569	61.9 (60.8–63.0)	< 0.001	< 0.001
Trade, certificate, diploma	1655	55.9 (54.1–57.7)		3445	65.0 (63.7–66.3)		< 0.001
Degree or higher	720	47.5 (45.0–50.0)		1548	51.0 (49.2–52.8)		0.03
Unable to save	2280	58.3 (56.8–59.9)	< 0.001	2764	63.2 (61.8–64.7)	.001	< 0.001
Can save	4033	53.6 (52.5–54.8)		6383	60.4 (59.5–61.4)		< 0.001
Obese	2270	19.2 (18.5–20.0)		4165	26.5 (25.8–27.2)		< 0.001
No schooling to secondary	1516	20.7 (19.8–21.7)	< 0.001	2085	28.2 (27.2–29.3)	< 0.001	< 0.001
Trade, certificate, diploma	555	18.8 (17.4–20.2)		1521	28.7 (27.5–30.0)		< 0.001
Degree or higher	198	13.1 (11.5–14.9)		557	18.3 (17.0–19.8)		< 0.001
Unable to save	911	23.3 (22.0–24.7)	< 0.001	1370	31.3 (30.0–32.7)	< 0.001	< 0.001
Can save	1300	17.3 (16.5–18.2)		2626	24.9 (24.1–25.7)		< 0.001
Sufficient fruit consumption	5026	39.7 (38.9–40.6)		7062	42.3 (41.6–43.1)		< 0.001
No schooling to secondary	3070	38.7 (37.6–39.8)	< 0.001	3224	40.6 (39.5–41.7)	< 0.001	0.02
Trade, certificate, diploma	1202	38.6 (36.9–40.3)		2302	41.1 (39.9–42.4)		0.02
Degree or higher	744	46.9 (44.4–49.4)		1526	48.7 (47.0–50.5)		0.23
Unable to save	1466	34.6 (33.2–36.0)	< 0.001	1653	35.6 (34.3–37.0)	< 0.001	0.31
Can save	3386	42.5 (41.4–43.6)		5024	45.1 (44.2–46.0)		< 0.001
Chronic Conditions							
Diabetes	875	6.9 (6.5–7.4)		1516	9.1 (8.6–9.5)		< 0.001
No schooling to secondary	609	7.7 (7.1–8.3)	< 0.001	822	10.3 (9.7–11.0)	< 0.001	< 0.001
Trade, certificate, diploma	184	5.9 (5.1–6.8)		507	9.1 (8.3–9.8)		< 0.001
Degree or higher	80	5.1 (4.1–6.2)		184	5.9 (5.1–6.7)		0.25
Unable to save	343	8.1 (7.3–9.0)	< 0.001	524	11.3 (10.4–12.2)	< 0.001	< 0.001
Can save	513	6.4 (5.9–7.0)		916	8.2 (7.7–8.7)		< 0.001
Current mental health condition	1987	15.7 (15.1–16.4)		3364	20.1 (19.5–20.7)		< 0.001
No schooling to secondary	1240	15.6 (14.8–16.4)	0.74	1659	20.8 (19.9–21.7)	< 0.001	< 0.001
Trade, certificate, diploma	501	16.1 (14.9–17.4)		1209	21.6 (20.5–22.7)		< 0.001
Degree or higher	243	15.3 (13.6–17.2)		494	15.8 (14.5–17.1)		0.68
Unable to save	1007	23.8 (22.5–25.1)	< 0.001	1411	30.3 (29.0–31.7)	< 0.001	< 0.001
Can save	918	11.5 (10.8–12.2)	< 0.001	1790	16.0 (15.4–16.7)		< 0.001
Psychological distress	1533	12.1 (11.5–12.7)		1678	10.1 (9.6–10.6)		< 0.001
No schooling to secondary	1058	13.3 (12.6–14.1)	< 0.001	841	10.7 (10.0–11.4)	< 0.001	< 0.001
Trade, certificate, diploma	318	10.2 (9.2–11.3)		633	11.3 (10.5–12.2)		0.11
Degree or higher	152	9.6 (8.2–11.1)		203	6.5 (5.7–7.4)		< 0.001
Unable to save	876	20.7 (19.5–21.9)	< 0.001	826	17.9 (16.8–19.0)	< 0.001	0.001
Can save	607	7.6 (7.0–8.2)		778	7.0 (6.5–7.5)		0.11
At least one Chronic Condition	5063	40.0 (39.2–40.9)		6664	39.8 (39.1–40.6)		0.73
No schooling to secondary	3407	42.9 (41.9–44.0)	< 0.001	3537	44.4 (43.3–45.5)	< 0.001	0.07
Trade, certificate, diploma	1175	37.8 (36.1–39.5)		2194	39.2 (37.9–40.4)		0.20
Degree or higher	471	29.7 (27.5–32.0)		924	29.5 (27.9–31.1)		0.91
Unable to save	1891	44.6 (43.1–46.1)	< 0.001	2133	45.8 (44.4–47.3)	< 0.001	0.26
Can save	3025	37.9 (36.9–39.0)		4189	37.6 (36.7–38.5)		0.60
Two or more Chronic Conditions	1538	12.2 (11.6–12.8)		2172	13.0 (12.4–13.6)		0.04
No schooling to secondary	1111	14.0 (13.3–14.8)	< 0.001	1326	16.6 (15.8–17.5)	< 0.001	0.001
Trade, certificate, diploma	320	10.3 (9.3–11.4)		639	11.4 (10.6–12.3)		0.11
Degree or higher	104	6.5 (5.4–7.9)		204	6.5 (5.7–7.4)		0.98
Unable to save	614	14.5 (13.5–15.6)	< 0.001	815	17.5 (16.5–18.6)	< 0.001	< 0.001
Can save	890	11.2 (10.5–11.9)		1240	11.1 (10.5–11.7)		0.92

Note ^a^ – p values are associated with chi-squared test of risk factor/chronic condition and educational attainment/household money situation for 2002/4 only; ^b^: *p* values are associated with chi-squared test of risk factor/chronic condition and educational attainment/household money situation for 2013/15 only; ^c^: p values are associated with chi-squared test for trend between two time points, 2002/4 and 2013/15 for corresponding risk factor/chronic condition, and for each category of educational attainment/household money situation by risk factor/chronic condition

The subjectively measured income related figures are confirmed by those of the ABS that has generally reported a steady increase in the mean real equalized disposable household income over this period (with the only exception of years around 2010 in which no increase was observed) [21]. As this increase was across all the income quartiles, income disparity does not seem to have increased in the last decade in SA.

**Fig. 1:**
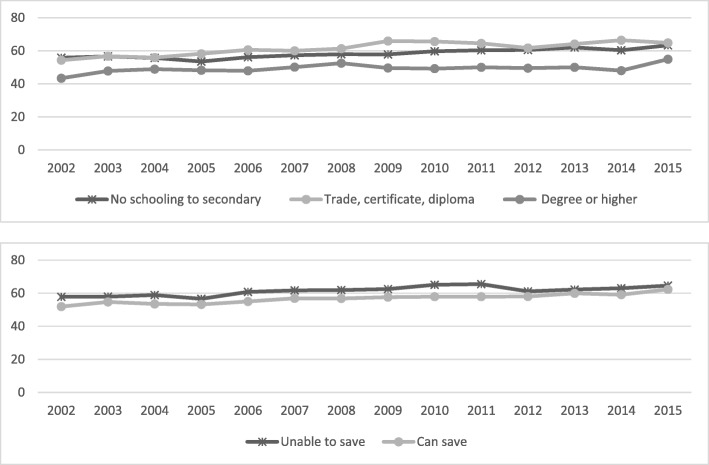
Annual prevalence, overweight and obesity by educational attainment and household money situation, 2002 to 2015

**Fig. 2:**
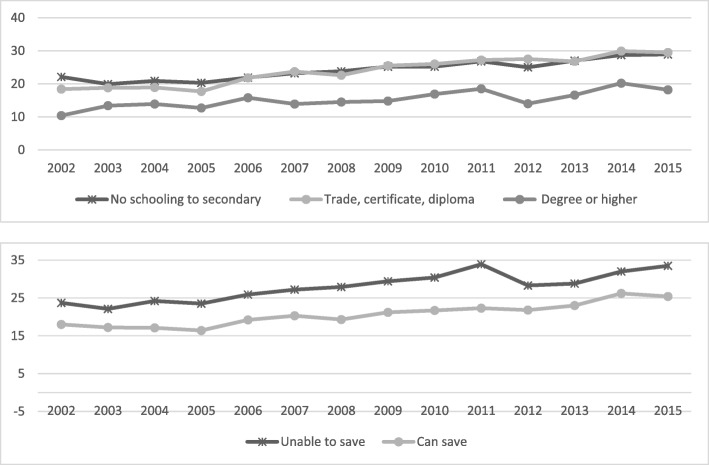
Annual prevalence, obese by educational attainment and household money situation, 2002 to 2015

**Fig. 3:**
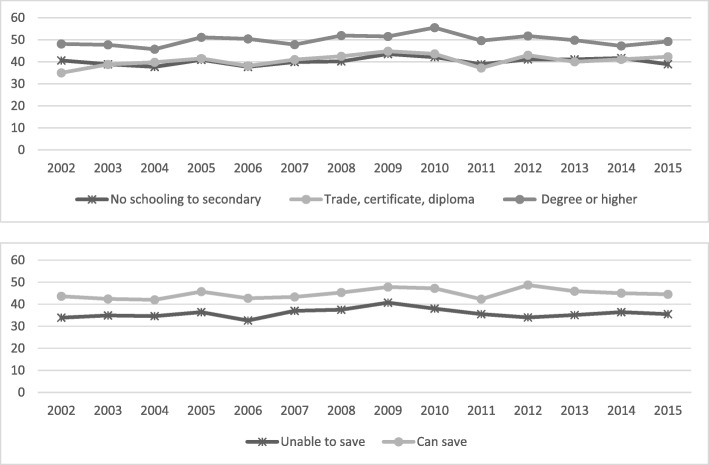
Annual prevalence, consuming sufficient amount of fruit daily (2 or more serves) by educational attainment and household money situation, 2002 to 2015

**Fig. 4:**
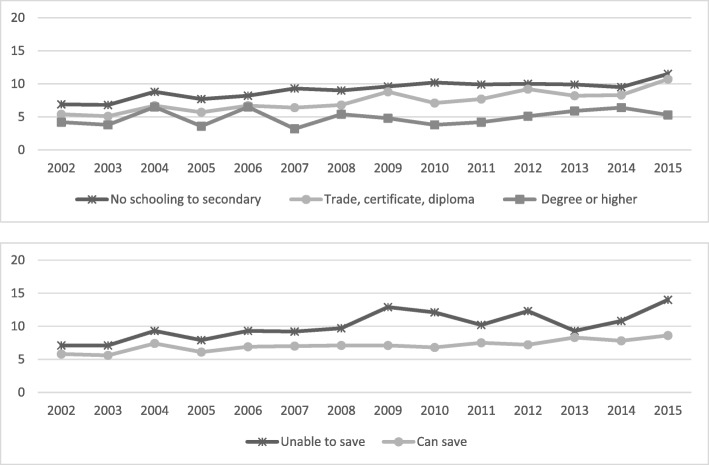
Annual prevalence, diagnosed diabetes by educational attainment and household money situation, 2002 to 2015

**Fig. 5:**
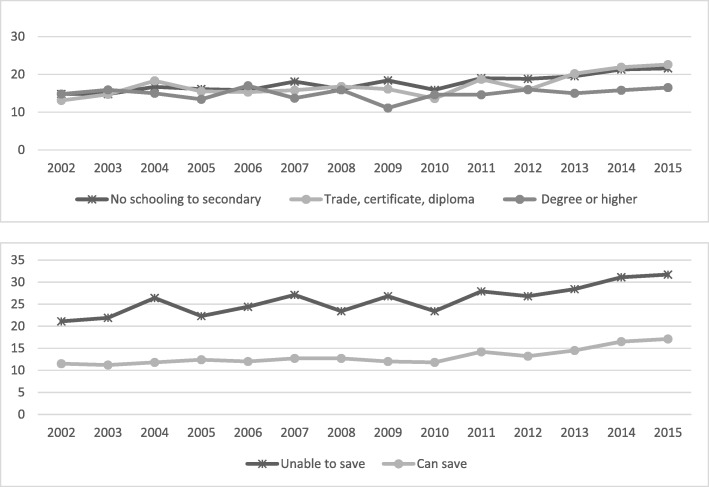
Annual prevalence, diagnosed mental health condition by educational attainment and household money situation, 2002 to 2015

**Fig. 6:**
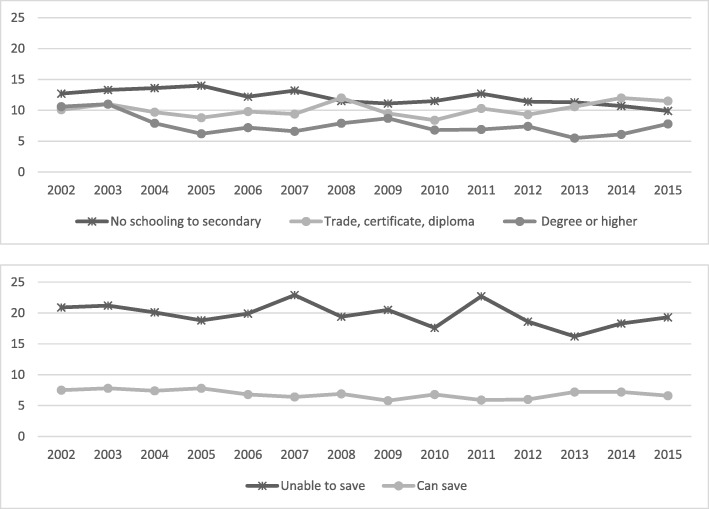
Annual prevalence, psychological distress by educational attainment and household money situation, 2002 to 2015

**Fig. 7:**
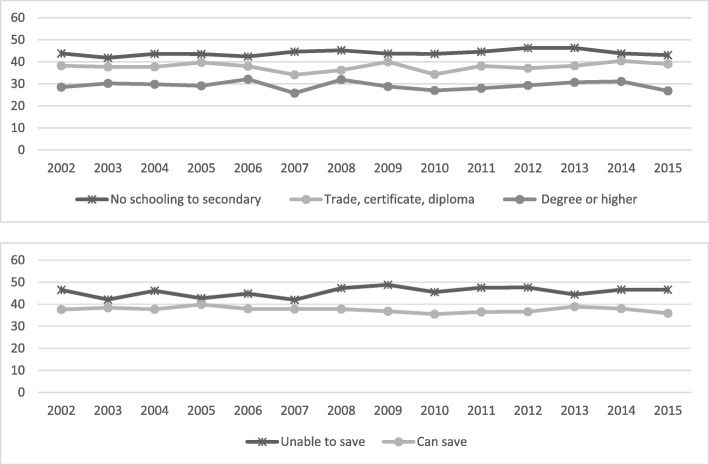
Annual prevalence, at least one chronic condition by educational attainment and household money situation, 2002 to 2015

**Fig. 8:**
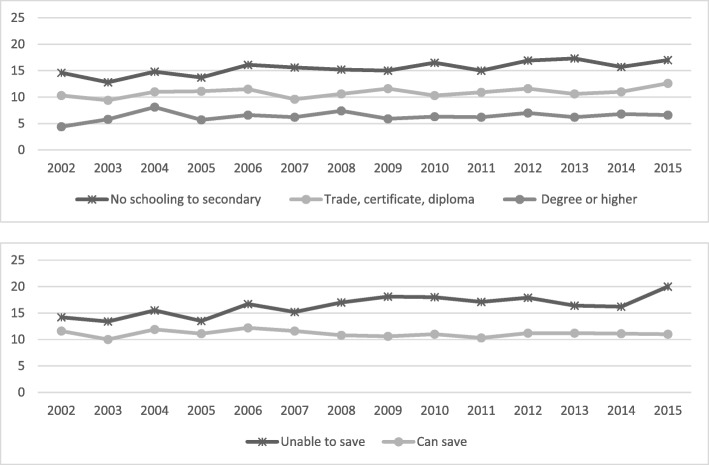
Annual prevalence, two or more chronic conditions by educational attainment and household money situation, 2002 to 2015

The analysis shows substantial differences in the prevalence of risk factors and chronic diseases among socio-economic subgroups (Table 1 and Figs. 1, 2, 3, 4, 5, 6, 7, 8). In regard to unhealthy weight, the overall increase over time is greater among the lower educated groups (*P* < 0.001), with a smaller increase seen for those with a university education. Similar results were seen for obesity, although there were increases across all social groups with the gap increasing over time.

In regard to fruit consumption, the higher educated group have changed little over the years, while in the other groups increases in consumption were apparent although starting from a lower base. There was an increase in fruit consumption for those who could save but lower estimates and no increase over time for those who cannot save, confirming a substantial inequality gap also for this variable.

The prevalence of diabetes, as expected, demonstrates differences among the lower social economic subgroups (*P* < 0.001) with the only subgroup that has not seen an increase over time being the higher educated group. Prevalence estimates continue to be lower for the higher social economic groups, and the gap in the other groups is increasing.

In regard to mental health conditions, the gap associated with education level increased over time, with the higher educated groups relatively stable while the other groups increased in prevalence (*P* < 0.001). In terms of psychological distress, the overall level decreased by 2% over time (*P* < 0.001) as did both the lowest and highest educated groups. Only a small decrease was shown among the ‘unable to save’ group, but this group is still far from closing a substantial gap in prevalence: 18% for the ‘unable to save’ group compared to 7% for the ‘can save’ group.

Differences in the prevalence of those with one chronic condition were relatively stable over time, with the expected (as highlighted by the other variables above) differences among socioeconomic groups. The differences are much more relevant when we consider those with two or more chronic conditions. The gap, again increasing over time, occurs as a result of the lower educated and those with lower economic conditions changing for the worse (*P* = 0.001 and *P* < 0.001) while the other groups are substantially stable over time.

## Discussion

SAMSS data have shown that health inequalities are stable, if not increasing, in SA with health disparities for lower educated and lower income groups, measured by those unable to save, appearing to be increasing in most of the health variables considered. Although it is not an aim of this manuscript to speculate about possible causes, which are quite often difficult to define [22], it is conceivable that most of these increases in inequality can be related to interventions not targeted to specific groups, or not specifically designed to be capable of reducing the gap [23–25]. Interventions targeted to the general population [23], such as, e.g., building cycling tracks (now covering most of the South Australian urban area), could be of benefit for those already active (typically higher educated, with higher income) and have no impact on those more vulnerable that are unable to buy a bike. This eventually produces an increase in the gap in the level of sedentary activity between classes. A call for more action and better understanding for more effectively targeting of the interventions is warranted. Staying with the biking example, this would mean health promotion activities involving more vulnerable communities. This could include, for example, offering free bikes, activities aimed to engage people in small bike tours, offering bikes to cycle to school and creating the conditions where this could easily happen.

It is evident, from the analysis presented in this study, the substantial role played by non-communicable disease and behavioural risk factor surveillance systems, in showing the evolutionary aspects of health disparities. Certainly, for these analyses the availability of a ‘real’ surveillance system [26, 27] rather than a few scattered surveys is fundamental. In our opinion, yearly or even with less frequently repeated surveys (the WHO suggest ‘at least every five years’) [28] provide little information when studying and showing trends. We believe that these analyses have only scratched the surface: much information can still to be obtained from surveillance data, particularly in understanding the mechanisms [29] which create health inequalities and, as we have seen, increase these inequalities. Specific analyses for sub-groups, defined on the basis of socio-demographic variables, but also geographically, can provide further information [30–32]. Data from surveillance systems, highlighted in this study, could be even more useful when linked with data from other sources (e.g. Census data), to study other potentially influencing social determinants such as social and cultural capital [33] or urban settings [34]. An even more important role in the future could have surveillance showing ‘what works’ in reducing health inequalities (when targeted interventions are implemented), given the potential use of these systems for evaluation purposes [4, 35, 36].

In this first paper we purposely limited the analyses so as to show simple time trends. Research is needed on these data, to better understand interaction of different social determinants of health, and the possible underlying mechanisms which creates and reinforces gaps. Certainly, for instance, the fact that over the years the number of those falling into more deprived groups has decreased in SA, due to the selective effect of social mobility, and could have left individuals in the lower strata individuals with characteristics that (directly or indirectly) induce worse health attitudes and behaviours.

However, using simple analyses to show how much the health inequality gap remains relevant, also creates several limitations. Some of these are related to the available data, and some are associate with the analysis conducted. A first weakness is the limiting of the risk factors assessed to two (BMI and fruit consumption) and three specific chronic conditions (diabetes, mental health and psychological distress). In addition, only two socio-economic related variables were used. Although the use of ‘ability to save’ as an indicator has been shown in Australia to be a valid indicator of financial security [37], it is acknowledged that other more reliable questions could have been used such as income. However, over the 10 year period, income earnings have increased for the whole of Australia which made it difficult to have comparable income groupings across the years. This study uses self-reported surveys which can potentially be subjected to bias due to socially desirable responses leading to possible over- or under-estimation of behaviors or health conditions, such as having a mental health condition or overweight and obesity due to incorrect reporting of height and weight [38]. However, these biases are of little importance if the aim of SAMSS is to study changes in the behaviour or health condition over time, assuming that the level of bias is constant over time.

The use of listed telephone numbers as the sampling frame can be considered a weakness of this study due to an increasing number of mobile-only households with the majority of these types of telephone numbers not being listed [39]. However, studies have shown that using this sample frame is still a viable source and reliable estimates can be produced when applying more effective weighting techniques, such as raked weighting [10], to overcome the sampling bias as well as non-response bias.

A further weakness is the lack of power in terms of data on Aboriginal status. In Australia, recent policy actions have focused on improving the health of Aboriginal populations with the Prime Minister of Australia in 2008 signing a statement committed to developing a long-term plan of action to end health inequalities between indigenous and non-indigenous populations [32]. Although SAMSS collects this information, the limited sample size does not permit analysis by Aboriginal status. It is also acknowledged that some of the increases in prevalence of mental health problems reported in this analysis could be the result of better diagnosing, which has been supported by additional funding from the Federal government in recent years. This could also impact our analyses by social class, leading to an underestimation of inequalities, since individuals who are more educated are also more likely to seek health care services and receive a diagnosis. The acknowledgment of public health campaigns in increasing the fruit consumption has also not been fully explored although earlier work with this surveillance system has shown promising results [35].

## Conclusions

It is evident, and this paper has contributed to providing more evidence, that there is much work still to do to “close the gap” [28, 40]. Also in more egalitarian societies with universal health systems, such as SA, more targeted and effective interventions are evidently needed to change the trends highlighted by SAMSS. BRFS can be a good source of information both to show the evolution of problems and to evaluate possible future interventions. Much effort is still required to ‘close the gap’ of health inequalities in SA. More precisely targeted and properly implemented interventions are needed.

## Additional file

Additional file 1: **Table S1.** Total sample size and median response rates, 2002 to 2015. Note: Response rates (RR1) were calculated from the final dispositions of the telephone numbers using the American Association for Public Opinion Research (AAPOR) [18] standard definitions. (DOCX 12 kb)

## Abbreviations

AAPOR RR1: American Association for Public Opinion Research Response Rate #1
ABS: Australian Bureau of statistics
BMI: Body mass index
CATI: Computer assisted telephone interviewing
EWP: Electronic white pages
HREC: Human research ethics committee
K10: Kessler 10
PROS: Population research & outcome studies
SA: South Australia
SAH: SA health
SAMSS: South Australian monitoring & surveillance system
WHO: World health organization
