# Supplementary material for: Increasing gaps in health inequalities related to non-communicable diseases in South Australia; implications towards behavioural risk factor surveillance systems to provide evidence for action
Source: BMC Public Health. 2019 Jan 8;19:37. doi: 10.1186/s12889-018-6323-7 (PMC6325833; doi:10.1186/s12889-018-6323-7)
Supplement: Supplementary file 1 — Table S1. Total sample size and median response rates, 2002 to 2015. Note: Response rates (RR1) were calculated from the final dispositions of the telephone numbers using the American Association for Public Opinion Research (AAPOR) [18] standard definitions. (DOCX 12 kb) [file 12889_2018_6323_MOESM1_ESM.docx]

| **Year** | **Total** | **RR1 (%)** |
| --- | --- | --- |
| 2002 | 3028 | 68.9 |
| 2003 | 6237 | 69.1 |
| 2004 | 7249 | 69.6 |
| 2005 | 7259 | 71.3 |
| 2006 | 7142 | 69.6 |
| 2007 | 7061 | 68.1 |
| 2008 | 7335 | 62.9 |
| 2009 | 7373 | 65.3 |
| 2010 | 7418 | 64.8 |
| 2011 | 7278 | 63.2 |
| 2012 | 7304 | 62.6 |
| 2013 | 7193 | 59.6 |
| 2014 | 7246 | 56.1 |
| 2015 | 3623 | 54.1 |
